# Supplementary material for: A common allele increases endometrial Wnt4 expression, with antagonistic implications for pregnancy, reproductive cancers, and endometriosis
Source: Nat Commun. 2024 Feb 12;15:1152. doi: 10.1038/s41467-024-45338-4 (PMC10861470; doi:10.1038/s41467-024-45338-4)

Supplemental Material: Figures

Figure S1. Design used for knock-in of the transgenic single nucleotide. rs3820282 is indicated in red with yellow highlighting the protospacer adjacent motif (PAM) and a box around the generated recognition sequence for restriction enzyme Tsp45I.

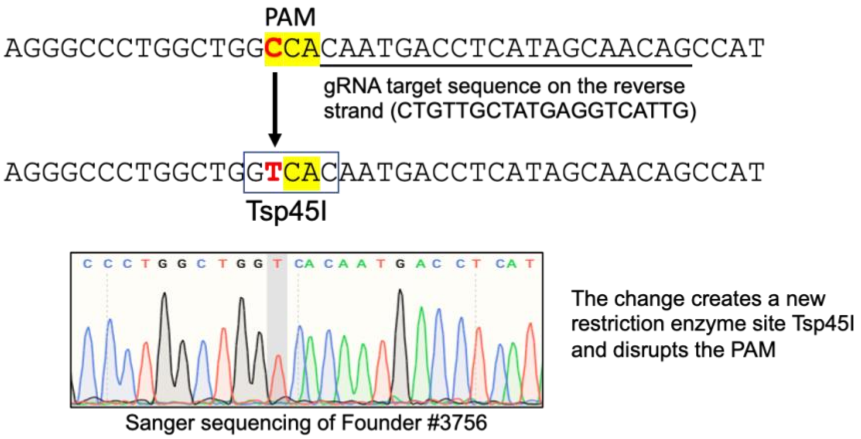

ssDNA donor sequence (asymmetric arm design)

TCCACATCACCTCAGTCTCCCAAGCTCTGGGTGGCTCTGACCCCAACGAACACACTCCTTTCCA
GTCTCCAGGGCCCTGGCTGGTCACAATGACCTCATAGCAACAGCCATTGGACACTCAGGGC

Figure S2. SNP does not influence ovarian expression of Wnt4 in the proestrus and estrus. Ovarian tissue from estrus and proestrus does not indicate significant upregulation ($P > 0.5$) of *Wnt4* expression in the ovary of TG (N=3 in proestrus, 5 in estrus) animals compared to WT (N=5 in proestrus, 3 in estrus). Statistical significance was evaluated by the Wilcoxon-Mann-Whitney rank sum test. Boxplots show the median (central line) and interquartile range (50% of the data fall within the box), with the whiskers covering the range of data (Q1 or Q3 + 1.5IQR).

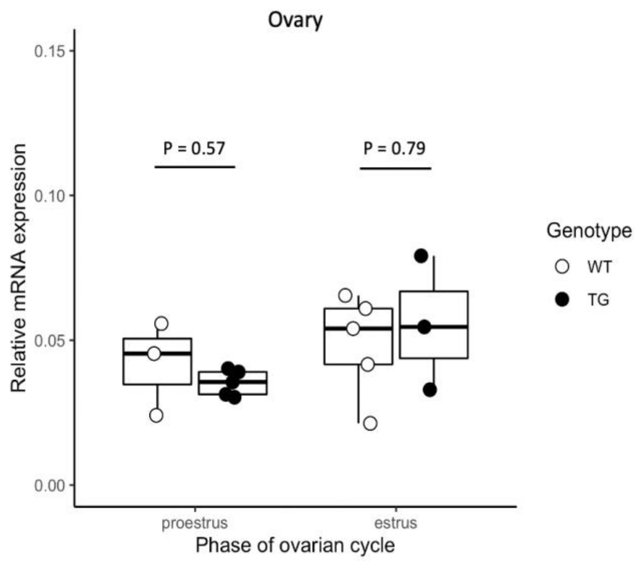

Figure S3. Immunocytochemistry of primary cell culture of wild type mouse endometrial stromal cells confirms the purity of extraction. Left: immunocytochemistry with anti-cytokeratin 7 (Ck7) antibody, marker of epithelial cells. Only a small number of cells was detected, less than 2%. Right: Vimentin (Vim) antibody, marker of stromal fibroblasts. The vast majority of cells were detected with this marker. All cells were also stained with DAPI to demonstrate the presence of non-AB-stained cells. Images represent three distinct wells of plated cells.

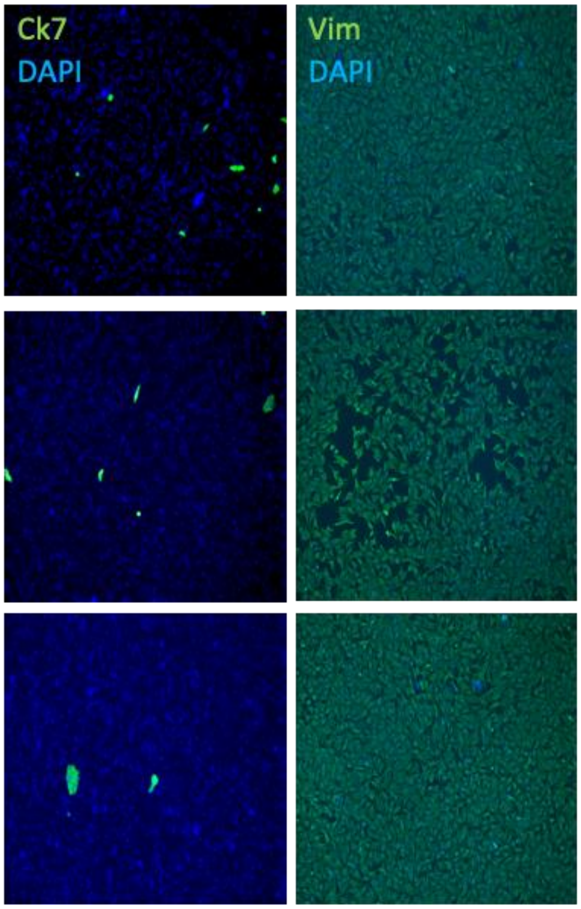

Figure S4. Reproducibility of proestrus uterine transcriptome. Animal genotype (TG or WT) corresponds to gene expression differences among samples following batch correction and normalization. A) The first principal component explained 68% variation and generally separated samples based on genotype. B) Pearson correlation between all samples was greater than 95% with hierarchical clustering distinguishing samples based on genotype.

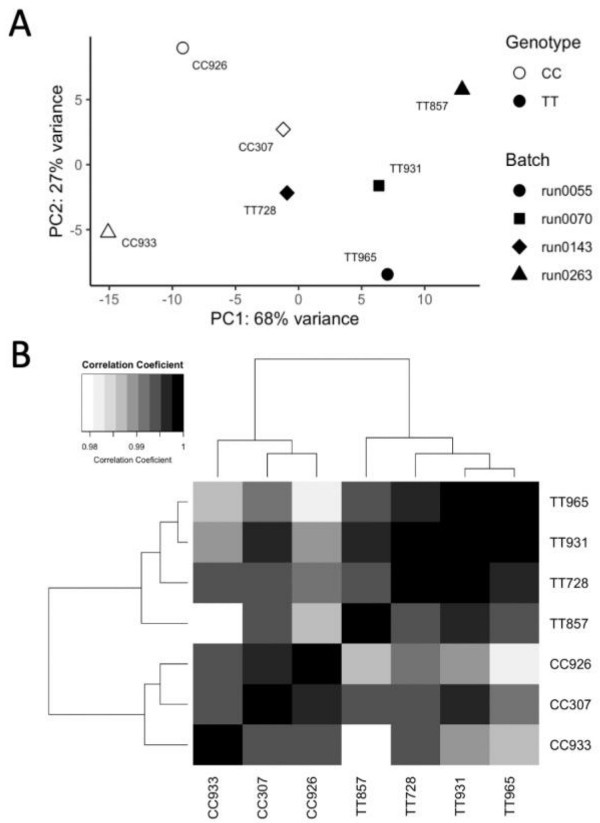

Fig S5. Distinct patterns of functional enrichment for differentially expressed genes. A) Gene set enrichment for Hallmark gene sets. Genes with decreased expression in the TG (i.e., higher expression in the WT) were enriched for functions associated with mitosis and cell cycle progression including E2F targets, G2M checkpoint, and Mitotic spindle. Genes with significantly increased expression in the transgenic line were enriched for a variety of functions including epithelial mesenchymal transition, hypoxia, myogenesis, and UV response down. B) Gene ontology over-representation in differentially expressed genes (top: biological process, middle: molecular function, bottom: cellular compartment) showed similar functional categories with chromosome organization and condensation overrepresented in WT whereas transporter activity and response to growth factor stimulus were overrepresented in TG.

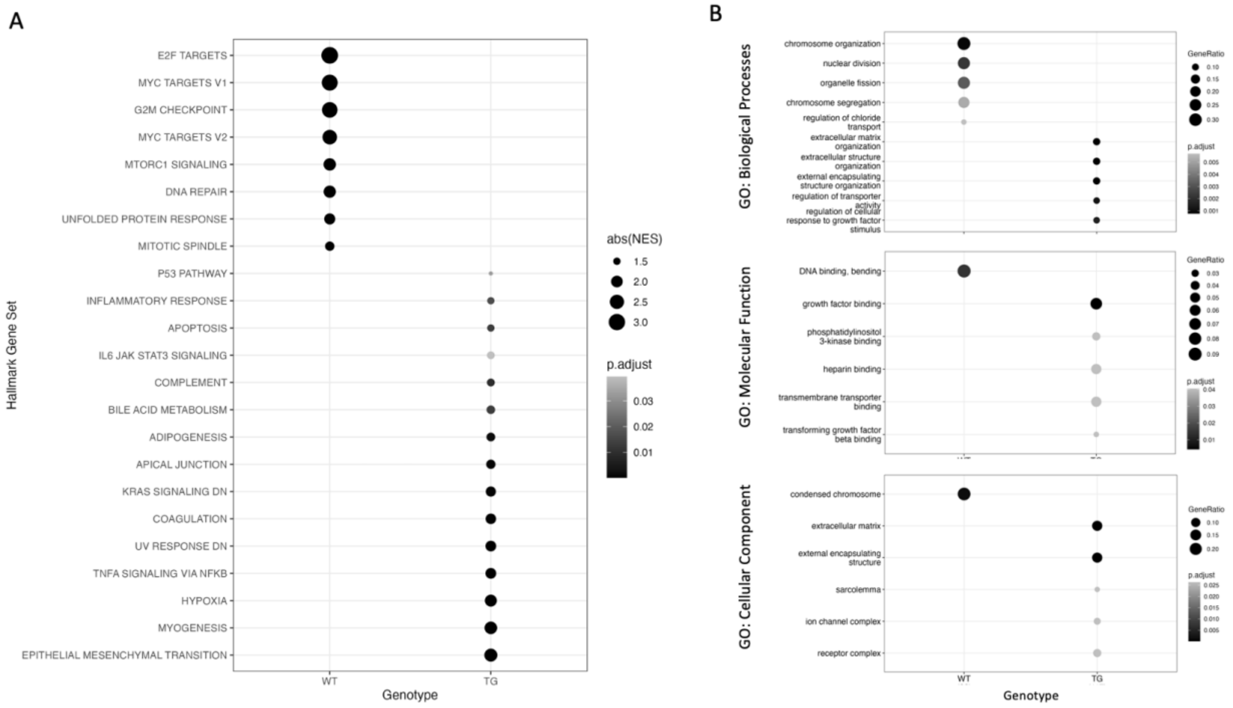

Figure S6: Relative expression of proliferation marker Mki67 in luminal epithelium of both genotypes. Expression was estimated from fluorescent immunohistochemically stained tissue. To avoid slide-specific background effects, the luminescence of luminal epithelium was assessed as differential luminescence relative to the glandular epithelium (non-specific signal in either genotype or timepoint). Two timepoints were analyzed: proestrus (PE) and estrus (E). We see decreased luminescence in transgenic relative to WT mice in proestrus (18.9 lum difference between genotypes, $P < 0.001$), and insubstantial difference between genotypes in estrus ($P > 0.05$).

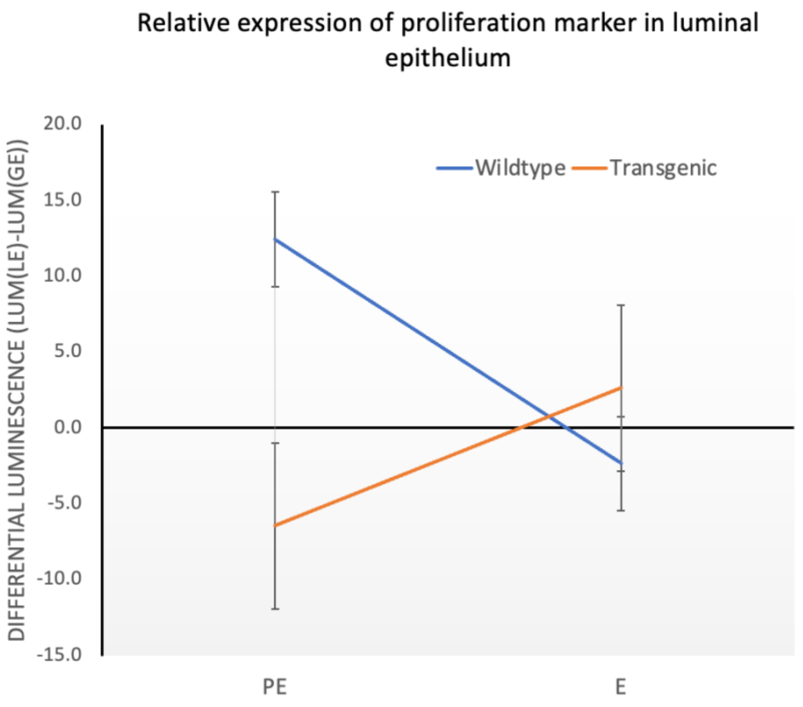

Figure S7. No difference in serum progesterone concentration. Systemic plasma samples collected immediately after cervical dislocation had no significant difference in progesterone concentration ($P = 1$). However, there was a greater range of concentrations in the TG (N=12) than the WT (N=5). Statistical significance was evaluated by the Wilcoxon-Mann-Whitney rank sum test. Boxplots show the median (central line) and interquartile range (50% of the data fall within the box), with the whiskers covering the range of data (Q1 or Q3 + 1.5IQR).

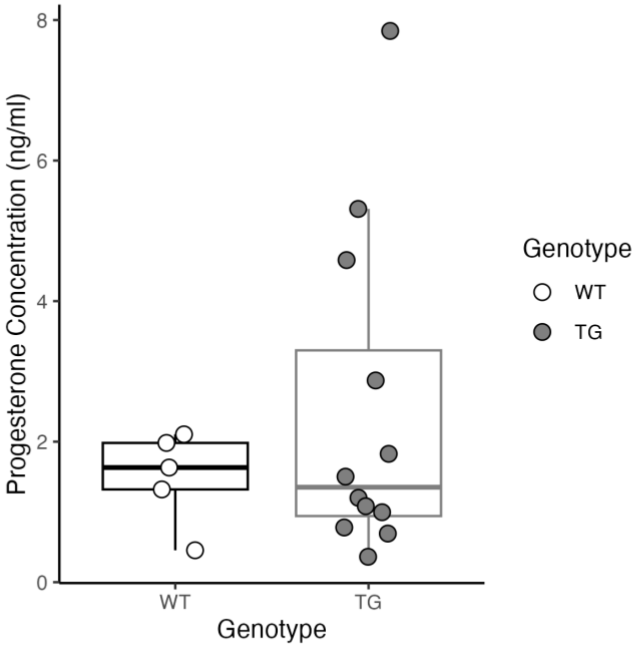

Figure S8. Support for non-canonical Wnt4 signaling. Immunohistochemistry using ß-catenin antibody in (A) proestrus and (B) estrus shows no translocation of ß-catenin into the nucleus in WT (left) or TG (right) uterus. Lack of ß-catenin translocation indicates that the canonical Wnt4 pathway is not activated. (C) Supporting the absence of canonical Wnt4 pathways, enhancer factor *Lef1* expression is at the limits of detection with insufficient expression to evaluate expression changes and high expression of *Axin2*, a member of the ß-catenin dissociation complex, with no evidence of increased expression associated with ß-catenin activation. (D) One of the non-canonical signaling pathways of Wnt4 is increased intracellular calcium which can result in phosphorylation of CamkII. Genes for the four alternative CamkII subunits were detected, with increased expression of *CamkIIb* in the TG (P = 0.007). Statistical significance was evaluated by the Wilcoxon-Mann-Whitney rank sum test. Boxplots show the median (central line) and interquartile range (50% of the data fall within the box), with the whiskers covering the range of data (Q1 or Q3 + 1.5IQR).

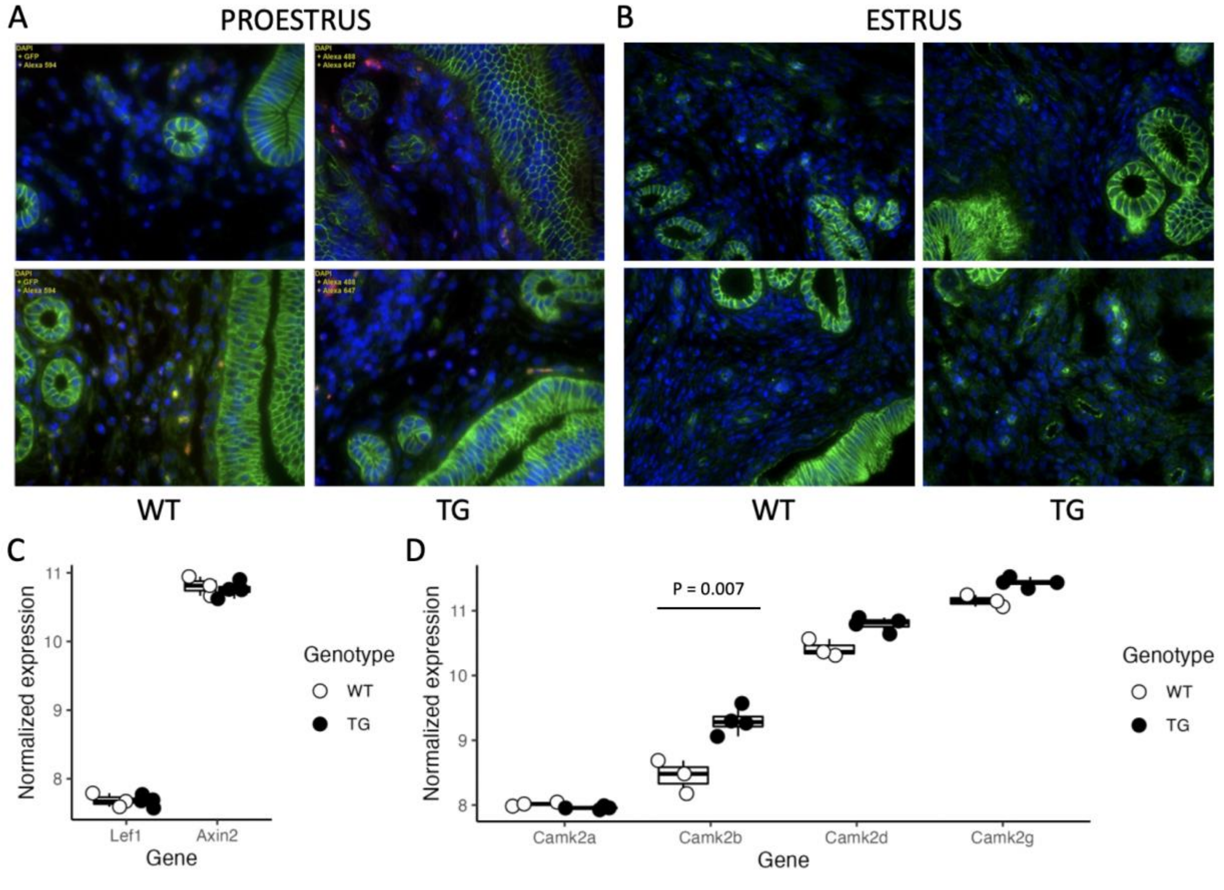

Figure S9. No significant difference in Wnt4 expression in the 7.5 dpc uterus or 17.5 dpc maternal-fetal interface. (A) No difference in *Wnt4* or *Cdc42* expression in the 7.5 dpc uterus between WT (N=3) and TG (N=3) animals (measured by qPCR). (B) *Wnt4* expression in the 17.5 dpc maternal-fetal interface was at the limits of detection with insufficient expression to evaluate expression changes and a trend towards decreased expression in the TG (=KI-1). No difference in *Cdc42* expression between WT (N=3) and TG (N=3) animals (measured by RNAseq). In both figures, statistical significance was evaluated by the Wilcoxon-Mann-Whitney rank sum test. Boxplots show the median (central line) and interquartile range (50% of the data fall within the box), with the whiskers covering the range of data (Q1 or Q3 + 1.5IQR).

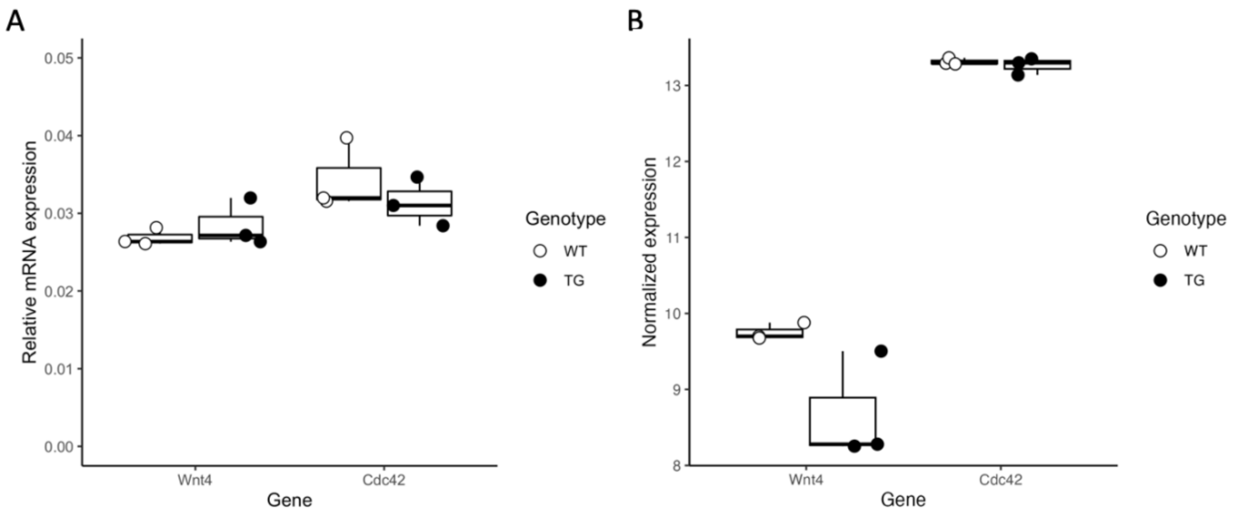

Figure S10. 17.5dpc maternal-fetal interface transcriptome. (A) Principal component analysis of normalized (variance stabilizing transformation) counts. The first principal component captured 75% of variance and generally corresponded to differences between WT (N=3) and TG (N=3) samples. (B) Volcano plot showing significance and effect size distribution. Log2 fold change values are calculated with respect to TG such that downregulated genes have a negative value and upregulated genes have a positive value. No genes met the adjusted P < 0.05 threshold for significant difference in expression. (C) Gene set enrichment for Hallmark gene sets revealed concentration of functional annotations related to epithelial to mesenchymal transition and myogenesis in genes with higher expression in TG.

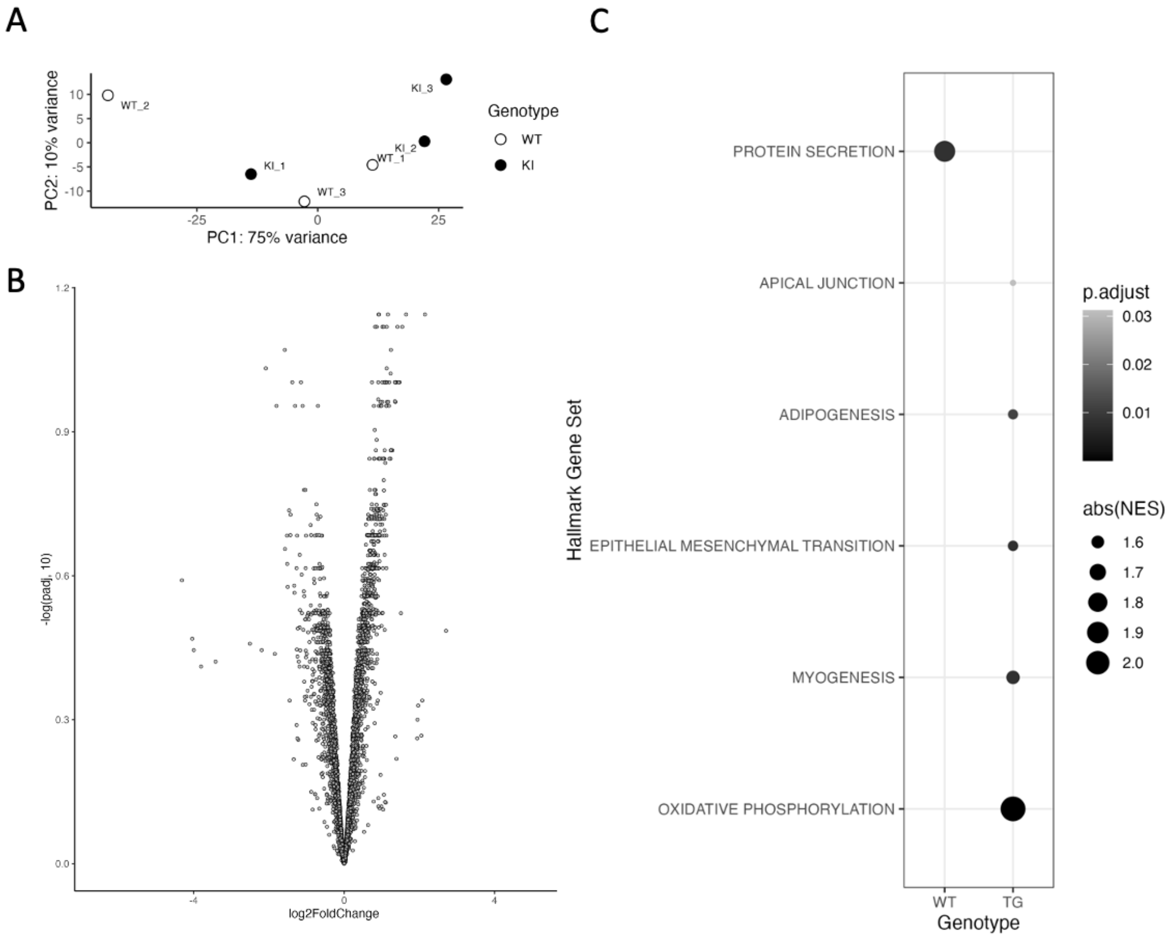

Figure S11. No differences in gestation length or litter size in transgenic lines. A) Gestation length and B) litter sizes were similar across all genotypes (P > 0.5). Independent individuals were assessed, with sample sizes 7 WT, 5 KI-1, 7 KI-2. Statistical significance was evaluated by the Wilcoxon-Mann-Whitney rank sum test. Boxplots show the median (central line) and interquartile range (50% of the data fall within the box), with the whiskers covering the range of data (Q1 or Q3 + 1.5IQR).

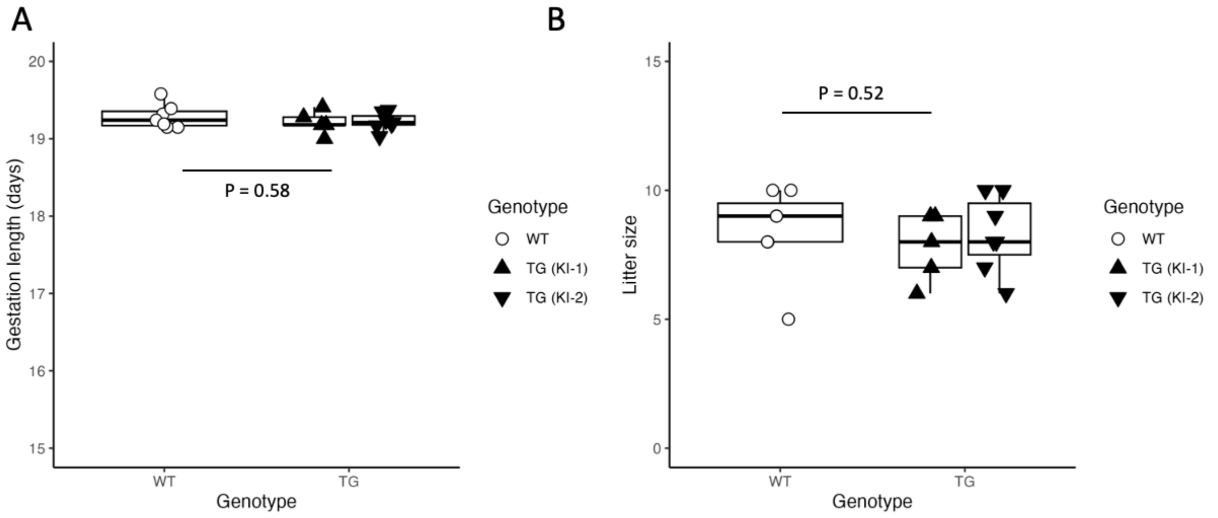

Figure S12. No genotype-specific accumulation of immune cells was detected in either estrus nor proestrus. Immunohistochemistry of estrus and proestrus uteri in cross section, stained with fluorescent-labeled antibodies against (A) Cd45, the pan-immune cell marker and (B) Cd11b, the marker of NK/ monocytes/ macrophages/neutrophils. Staining of immune cells was similar in both the TG and WT.

A. **B.**

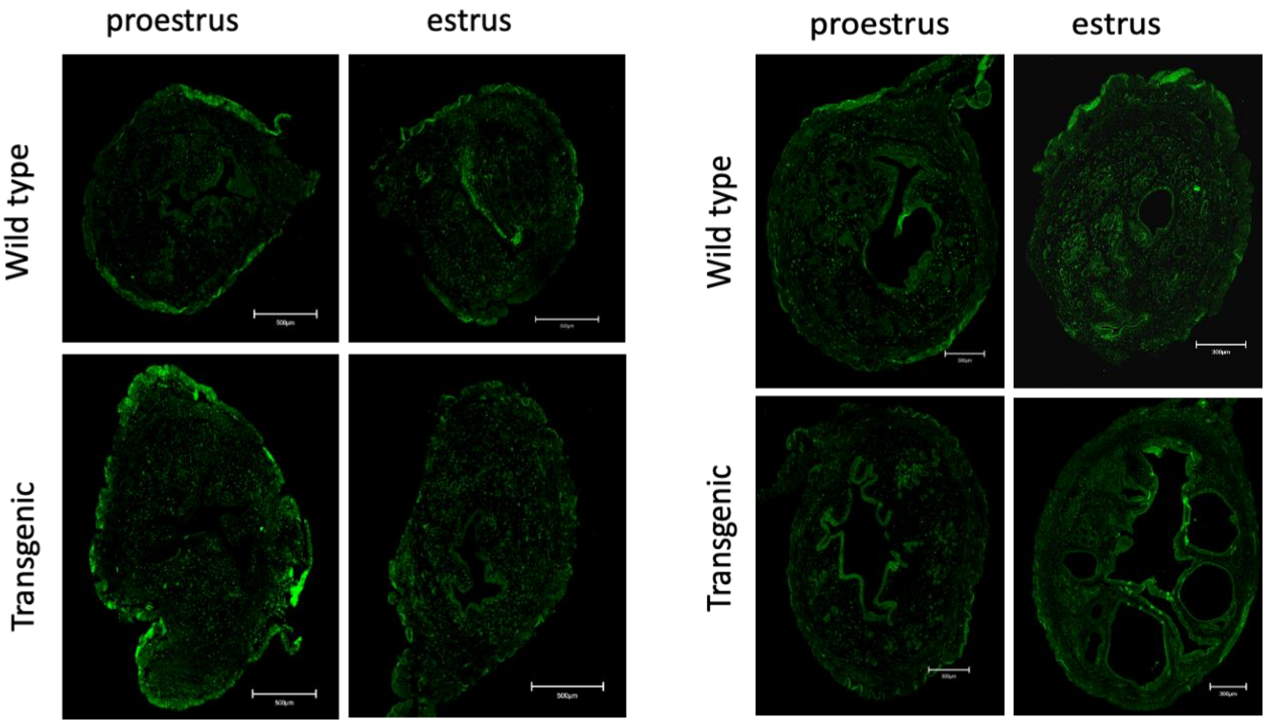

Supplement: Supplementary file 1 — Supplementary Information [file 41467_2024_45338_MOESM1_ESM.pdf]
