# Supplementary material for: A common allele increases endometrial Wnt4 expression, with antagonistic implications for pregnancy, reproductive cancers, and endometriosis
Source: Nat Commun. 2024 Feb 12;15:1152. doi: 10.1038/s41467-024-45338-4 (PMC10861470; doi:10.1038/s41467-024-45338-4)
Supplement: Supplementary file 3 — Description of Additional Supplementary Files [file 41467_2024_45338_MOESM3_ESM.pdf]

Description of Additional Supplementary Files

File Name: Supplementary Data 1
Description: All QPCR expression measurements obtained in the manuscript (normalized relative to GAPDH expression).

File Name: Supplementary Data 2
Description: The RNA seq expression levels of the proestrus and 17.5dpc (pregnancy) stage of both genotypes.

File Name: Supplementary Data 3
Description: Hallmark and Gene Ontology analyses of the RNA Seq data.

File Name: Supplementary Data 4
Description: Raw measurements of phenotypic data of both genotypes: Gestation length, Litter size, Image analysis of expression MKI67, uterine size, progesterone and PAS quantification.
